# Intraoperative Touch Imprint Cytology of Brain Neoplasms: A Useful High-Diagnostic Tool in 93 Consecutive Cases; Differential Diagnoses, Pitfalls, and Traps

**DOI:** 10.1155/2024/2346092

**Published:** 2024-01-04

**Authors:** Ali Koyuncuer

**Affiliations:** Department of Pathology, Umraniye Training and Research Hospital, University of Health Sciences, Istanbul, Türkiye

## Abstract

**Introduction:**

Intraoperative cytological examination of central nervous system (CNS) lesions was first introduced in 1920 by Eisenhardt and Cushing for rapid evaluation of neurosurgical specimens and to guide surgical treatment. It is recognized that this method not only confirms the adequacy of biopsy in CNS samples but also indicates the presence and preliminary diagnosis of lesional tissue.

**Methods:**

A total of 93 patients who underwent touch imprint cytology (TIC) for CNS tumors or lesions between 2018 and 2023 were included in the study. All cases were correlated with the final histopathological diagnosis, and pitfalls and difficulties encountered with discrepancies were noted.

**Result:**

The most common primary CNS tumors were gliomas and meningiomas, while secondary (metastatic) tumors were predominantly lung, breast, and gastrointestinal system carcinomas. Sensitivity, specificity, positive predictive value, and negative predictive value for diagnosis with TIC were 94.1%, 100%, and 61.5%, respectively. Final histopathological diagnosis by TIC was made in 88 cases (94.6%) and the discrepancy was found in 5 cases (5.37%). Three of the five discrepancies (3.2%) were haematolymphoid malignancies (two lymphomas and one plasma cell neoplasia), one glioblastoma, and one hemangioblastoma case.

**Conclusion:**

TIC is a fast, safe, and inexpensive diagnostic tool used during intraoperative neuropathology consultation. Awareness of the pitfalls of using this method during intraoperative consultation will enable high-diagnostic accuracy.

## 1. Introduction

The use of rapid diagnostic techniques in intraoperative consultation is the pathologist's main concern in the evaluation of neurosurgical specimens. This process begins with the differentiation of neoplastic from nonneoplastic, then primary from metastatic, and finally glial from nonglial[1]. Particularly in the case of small biopsies, the examination of stereotactic biopsies alone, without frozen section or cytological evaluation, has been shown to significantly reduce the diagnosis rate, making the preliminary evaluation of intraoperative cytological evaluation almost mandatory in the examination of neurosurgical specimens [2]. However, these specimens may have various limitations; among the nonpathologist factors, the fact that the specimen sent for intraoperative consultation does not represent the lesion and consists of fibrous tissue or necrosis affects the diagnosis, while the inexperience of the pathologist is the main factor in the correct diagnosis and evaluation [3]. Although the accurate diagnosis rate of both touch imprint cytology (TIC) and smears is around 90% in some studies, TIC cannot be performed in many medical centers because of the belief that frozen sections contain too much information and for financial reasons [4–6]. Although radio-image methods including intraoperative imaging have increased and improved today, TIC continues to be a very important diagnostic tool, especially in tumor surgery. The fact that the discrepancy rate for frozen section analysis is 3%, is indicative of the importance of TIC [7, 8]. In our study, it is aimed to determine the diagnostic pitfalls that are met by comparing the final paraffin section diagnoses of the cases we decided on in the intraoperative consultation with TIC and to identify important clues that will facilitate the approach to such cases, aiming to increase the importance of education and awareness about TIC.

## 2. Materials and Methods

Our study was conducted on neurosurgery specimens sent to the Pathology Department for intraoperative consultation between January 2018 and June 2023. This present study was approved by the Institute's Ethical Committee (Reference no: B.10.1.TKH.4.34.GP.0.01/92). We followed Helsinki's Ethical guidelines. A total of 144 cases operated on for intracranial masses were included, but arteriovenous malformations, intracranial hemorrhage and infection, or inflammatory conditions affecting both the dura and the brain parenchyma were not included in the study, but two case of hydatid cyst and gliosis that mimicked neoplasms and had a radiological mass image, and therefore had an intraoperative frozen section, were included in the study.

### 2.1. Procedure

Intraoperative consultation slides, TIC slides, squash smears, hematoxylin–eosin (HE)-stained histological paraffin sections, and all immunohistochemical slides from cases were retrieved and evaluated. TIC was used for all samples for which intraoperative consultation was performed because of an intracranial lesion. This was carried out by touching and rolling a small tissue biopsy on a glass slide. This technique can also be referred to as the drag, roll or touch, and pick procedure [9]. In this method, the short axis of the slide surface is used and slight pressure is applied to the tissue without crushing it. A small piece of the same specimen is then used for squash preparation (SP). In this method, the tissue is placed between two microscope slides, the slides are moved in opposite directions and the tissue is compressed between the two slides. Tissue fragments remaining on the slides are removed from the slide with small pincette forceps and stored for paraffin embedding. All TIC and SP specimens were rapidly fixed in 95% ethanol (ethanol) without drying and stained using the rapid HE-staining method. In some cases, we also evaluated the diagnostic superiority of the stains by staining with the rapid Papanicolaou stain. All intraoperative neuropathological consultation specimens were interpreted with TIC and SP only, simultaneous frozen section was not performed in our cases. Immunohistochemistry (Benchmark XT Ventanna; Ventana Medical Systems, Tucson, AZ) was then performed on formalin-fixed paraffin-embedded sections.

### 2.2. Cytological Diagnosis Evaluation and Approach

It is necessary to determine whether the material obtained intraoperatively is a biopsy or a resection, and to report that this tissue is a “lesional tissue” with a diagnosis. A very strict cytological diagnosis or grading is unnecessary and can sometimes lead to irreversible major surgical procedures. Therefore, the most important point is to know that the biopsy is representative of the lesion. If the sent biopsy contains a lesion and is very small, additional tissue should be sent for further immunohistochemical, molecular genetic, and sometimes microbiological, inflammatory studies, and the adequacy of the biopsy should be reported. In this consultation, the pathologist should make every effort to recognize neoplasms such as infections that do not require total resection, inflammatory lesions such as multiple sclerosis (MS), lymphoma, germ cell tumors, langerhans cell histiocytosis, and small cell carcinoma. These lesions or tumors can be treated with chemoradiotherapy. Although it is not always possible to differentiate between gliomas and glioneural tumors in the cytological preparations, the pathologist should be aware that resection is the preferred condition for infiltrative gliomas. Fibrillar processes, cytoplasmic details, and nuclear cytomorphological features are quite well-recognizable in alcohol-fixed cytological preparations in the absence of drying artifacts [8]. In the central nervous system (CNS) intraoperative consultations, touch imprint should be evaluated in the first step in cytology during four main features: nuclear, cytoplasmic, background, and architectural features [10–12]. Irrespective of the type of material presented to the intraoperative consultation, the basic cytological parameters to be evaluated at the first microscopic evaluation were determined algorithmically to support the diagnosis. Nuclear features evaluated included shape, hyperchromatism status, anaplasia, pleomorphism, mitotic figures, nuclear membrane borders, presence and appearance of the nucleolus, and pseudoconclusions. Cytoplasmic features include processes, vacuolization status, presence of granularity, and amount or absence of cytoplasm. Among the background features, fibrillarity, necrosis, rosenthal fibrils, psammoma bodies, reticulin or collagen fibers, myxoid matrix, cellular debris, and cellular infiltrates were investigated and evaluated. Among the architectural features, whorls, papillae and rosettes, or fascicles formations were investigated [12–14].

### 2.3. Histopathological Evaluation

All HE slides and immunohistochemical slides were reevaluated, and primary CNS tumors were recategorized according to the World Health Organization's fifth edition classification published in 2021. This edition considers significant changes to both CNS tumor terminology and integrated and layered reporting [15, 16]. Our study not only confirmed the presence of neoplasia but also tried to determine the tumor subtype and histological grade as accurately as possible. For the grade evaluation, ±1 grade change was recorded and the correlation was evaluated.

### 2.4. Statistical Analysis

Statistical analysis was performed using SPSS 22 (SPSS Statistics for Windows; IBM, Armonk, New York, USA). While Fisher's exact test was mainly used in the tables, statistical differences between groups were evaluated using the Mann–Whitney *U* test, or all associations between categorical data were assessed using *χ*^2^ tests. Fisher's exact test, and the median and standard deviations for variables such as age and gender. Sensitivity, specificity, and positive and negative predictive values (PPV and NPV) were calculated and interpreted. *P*-values < 0.05 were considered as significant.

## 3. Results

The mean age of the patients was 51.4 years and the predominant age range was 60–75 years. Male to female ratio was 1.4/1, the earliest age was 2 years and the oldest patient was 83-year old. Intraoperative consultation was performed in 64.6% of the cases. The most common lesion location was right cerebral lobe in 25 cases (17.4%). The most common glial tumor was Glioblastoma, IDH-wild type, Grade 4; 24 cases (16.7%), meningiomas were 22 cases (15.3%), metastatic tumors were 19 cases (13.2%) (Table 1). The accuracy rate of TIC was 94.6% (Tables 2–4). Among primary CNS tumors, the best grade correlation occurred in high-grade gliomas. In 94.7% of the 19, Grades 3 and 4 glial tumors, the grade was compatible with the final diagnosis. The correlation was complete in pilocytic astrocytomas and low-grade astrocytomas. In Grade 1 meningiomas, full correlation with the final diagnosis was achieved, but in atypical meningiomas, harmony was achieved in terms of cellularity, but grade agreement decreased because sufficient areas of mitosis and necrosis were not observed. Sensitivity, specificity, PPV, and NPV of TIC for detection of glial, nonglial, and metastatic tumors were 94.1%, 100%, 100%, and 61.5%, respectively. The discordance between imprint cytology and final histopathological diagnosis was one patient with plasma cell neoplasia which could not be diagnosed because the specimen sent for intraoperative consultation was crushed, two patients with lymphoma diagnosed as atypical lymphoid proliferation, one patient with glioblastoma with a tumor-free specimen, and one case of hemangioblastoma with insufficient cells. Although all of the discrepancies were due to reasons independent of the pathologist, we included these two patients in the discrepancy group because there may be those who prefer a direct diagnosis of lymphoma, although the intraoperative diagnosis of “atypical lymphoid proliferation” with TIC is considered sufficient for haematolymphoid malignancies. We also included the responsibility of the surgeon who sent the nonneoplastic tissue and a fragile piece of stromal tissue in the discrepancy group.

## 4. Discussion

In neuropathology practice, intraoperative consultation shows some differences from other branches because it contains pitfalls, hidden pitfalls, and sometimes mistakes. One of the main reasons for this may be related to the fact that CNS tumor operations are not performed at the same level in every hospital, the low case rate, and the poor familiarity and education of the pathologists in this regard. Neoplastic and nonneoplastic lesions with overlapping pathological changes are common [17]. The low number of neuropathologists and less transfer of experience are other reasons for the low number of pathologists in intraoperative consultation. However, for pathologists who have not received adequate neuropathology training, such specimens, especially intraoperative ones, can be annoying and even intimidating. One of the most important purposes of intraoperative consultation is to quickly reach a pathological diagnosis and provide accurate guidance to neurosurgeons in determining the course of surgery. Although it varies depending on the type of surgery, the most important issue after diagnostic adequacy is to know the presence of tissue in the lesion, and in case of the prevalence of inflammatory cells, a warning should be made for microbiological examinations or cultures in terms of infectious agents, inflammatory processes, and demyelinated diseases. For neoplastic lesions, it is important to define the tumor type, considering their sensitivity to chemotherapy or radiotherapy. In terms of lesions that do not require gross total resection, lymphoma, germ cell tumors, langerhans cell histiocytosis, and small cell carcinoma can be counted among these. However, if some infiltrating gliomas are recognized, total resection is recommended whenever possible; these tumors include pilocytic astrocytoma, ependymoma, pleomorphic xanthoastrocytoma, and ganglioglioma. A tumor showing radiological contrast enhancement can give us an idea about the histological grade. If an infiltrating glioma is present, it is most likely a high-grade glioma. Necrosis, mitotic figures, and vascular endothelial proliferation should be looked for. At such times, especially during gross examination of the specimen, taking samples from gray–white transition areas and areas rich in vessels other than hemorrhage makes it easier to identify a high-grade tumor. One of the most important diagnostic issues, especially in Grade 2 infiltrative gliomas, is the cellularity of the specimen. While the presence of atypical cells within the diffuse glial fibrillary process leads to the diagnosis of infiltrating astrocytoma, the high frequency of mitotic figures should raise suspicion for Grades 3 or 4 tumors. Necrosis or microvascular proliferation is especially significant for a high-grade tumor such as glioblastoma [8]. Among the pitfalls we encountered, were difficulties in detecting and counting mitotic figures. Another trap is the obvious interpretation of nuclear details. These two situations required significant effort and time. The main purpose of this retrospective study was to determine the diagnostic role and diagnostic accuracy of TIC alone on intraoperative neurosurgical specimens in a tertiary care teaching and research hospital, and our results showed a high-accuracy rate compared to the literature, although there are few reported studies.

**Figure 1:**
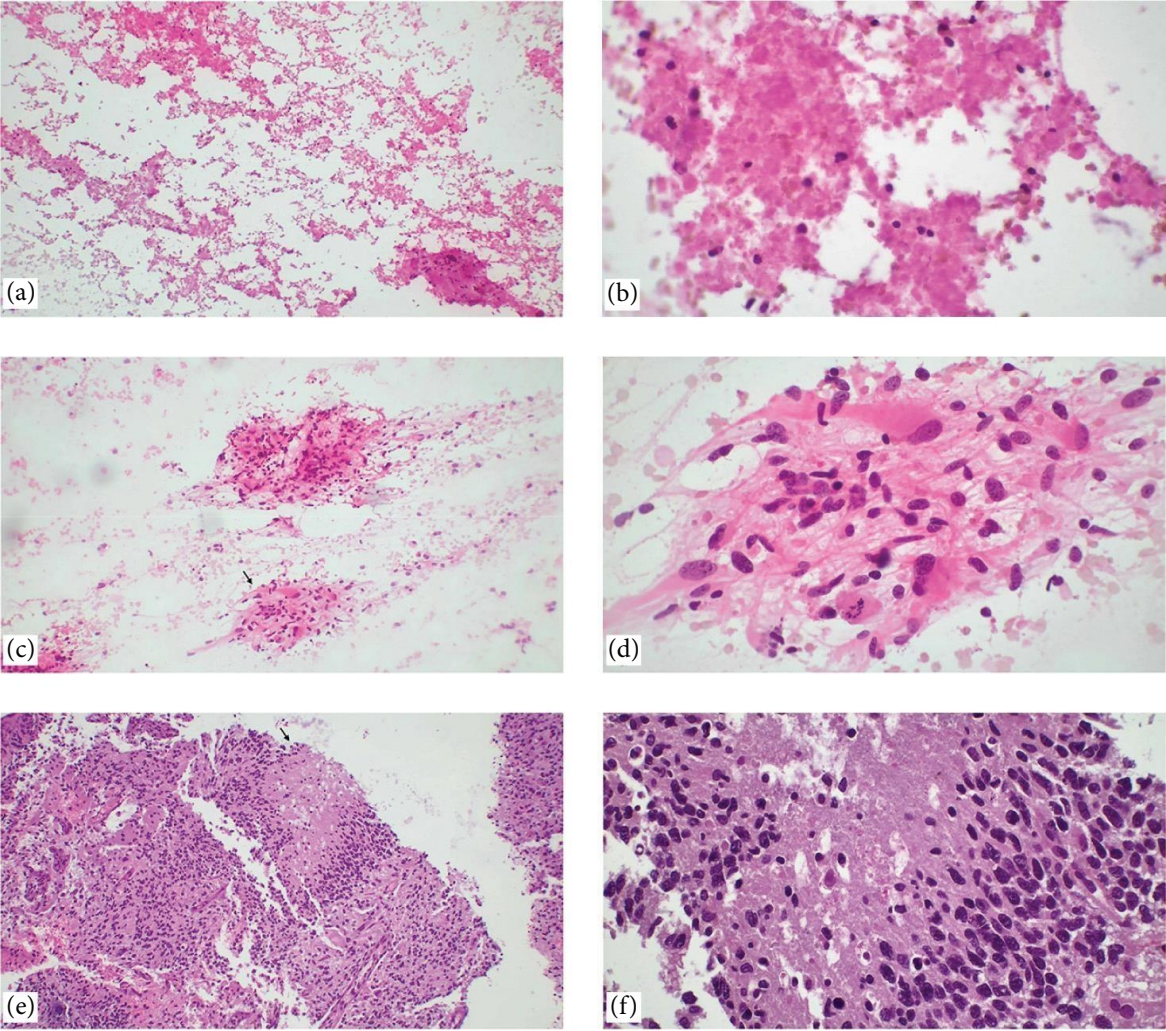
Glioblastoma. (a, b) If only necrosis is detected in an intraoperative evaluation, which is defined as a nonspecific pattern in touch imprint cytology, the surgeon is requested to send a shallower specimen with a high probability of containing viable tumor cells for correct classification (H&E, ×40, ×400). With the resampling by the surgeon, (c, d) touch imprint cytology, and (e, f) histopathological sections show marked atypia, microvascular proliferation, mitotic figures, pseudopalisating necrosis, and cellular pleomorphism (H&E, ×40, ×100, ×400).

**Figure 2:**
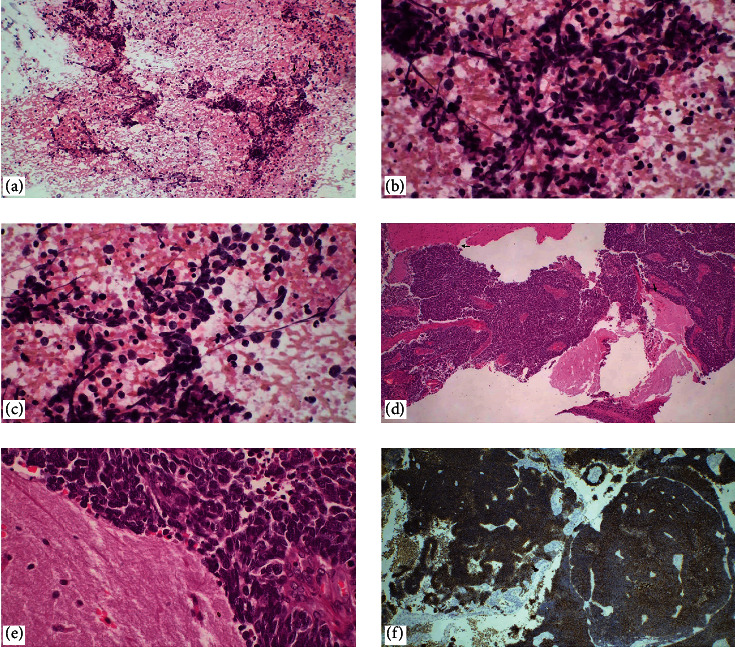
Metastatic small-cell carcinoma of the lung. (a–c) In touch imprint cytology, hypercellular necrotic smears, hyperchromatic granular chromatin, and oval-shaped cells with high-nucleus cytoplasm ratio, crush artifact (H&E, ×100, ×400), (d, e) small-cell carcinoma metastasizing to the brain parenchyma. In a compact growth pattern, a sharp demarcation between the carcinoma and the brain parenchyma is observed (H&E, ×40, ×400), (f) CD56 is diffusely positive in immunohistochemical studies (×40).

**Figure 3:**
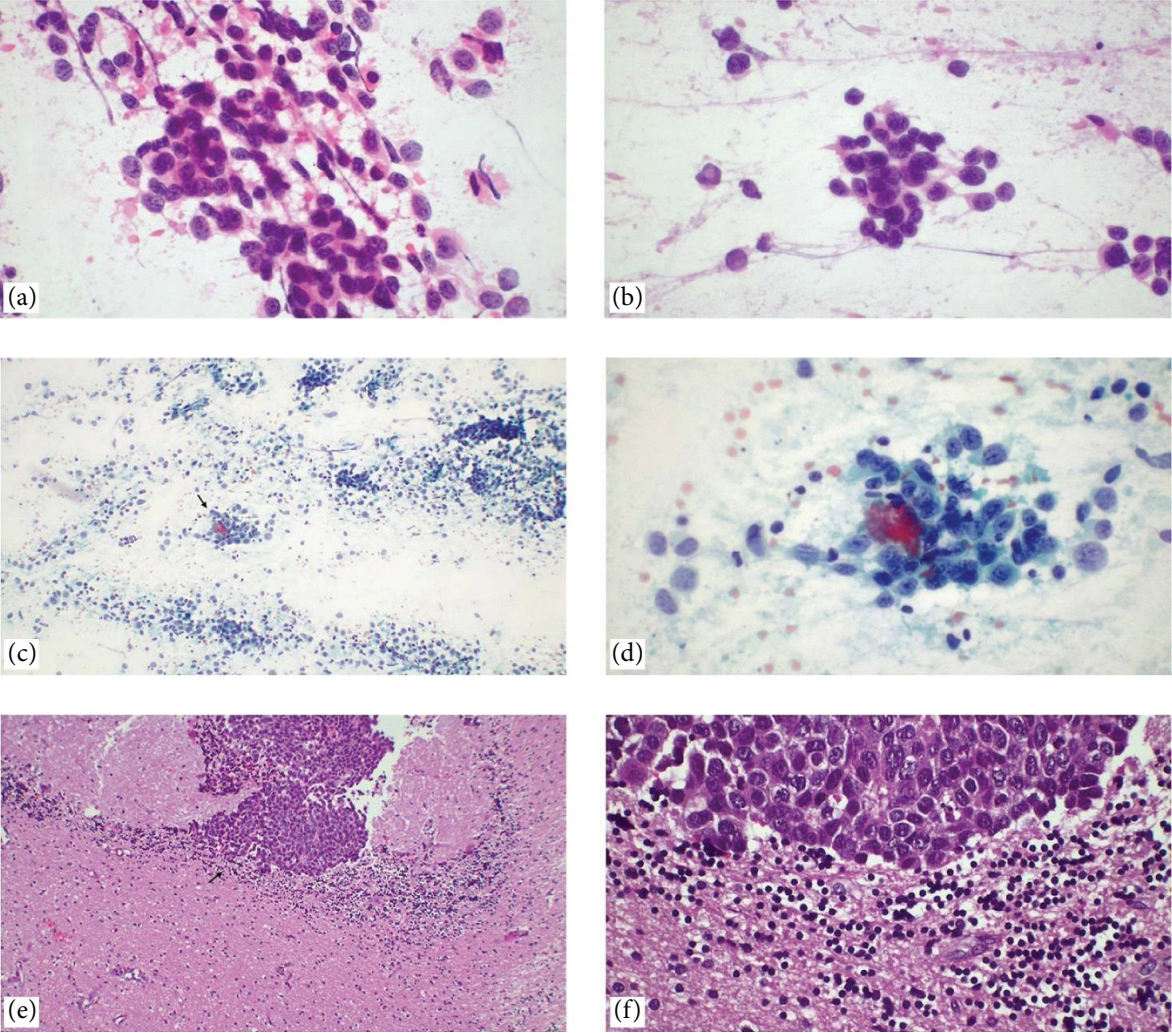
Metastatic breast carcinoma. In touch imprint cytology, highly cellular, of various shapes and sizes, with visible nucleoli, eccentrically located, hyperchromatic oval nuclei (a, b) H&E, ×400, (c, d) Papanicolaou stain, ×100, ×400. (e, f) paraffin section of the case diagnosed as metastatic breast carcinoma, NOS. A sharp demarcation between the carcinoma and the brain parenchyma is observed (H&E, ×100, ×400).

**Figure 4:**
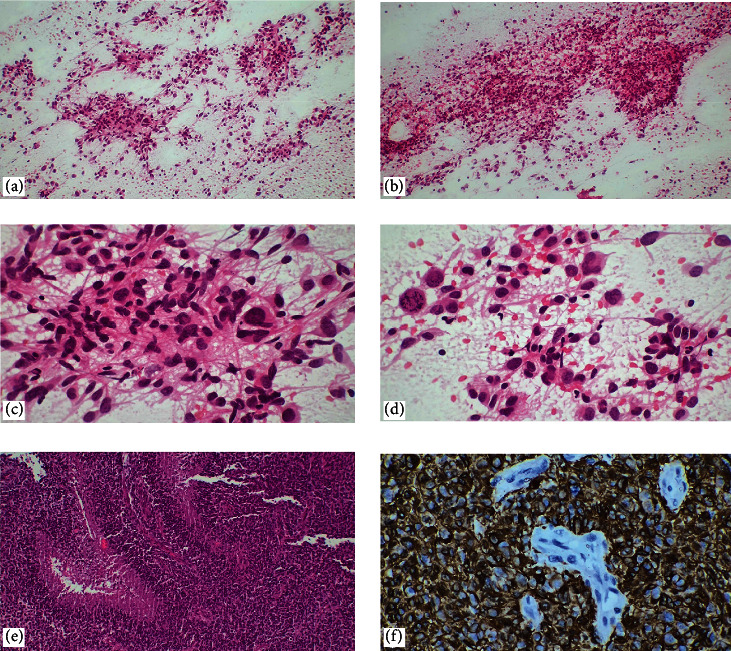
Glioblastoma. (a–d) Touch imprint cytology shows typical cytological features of high-grade glioma. Fibrillary, bloody necrotic background and some spindled, epithelioid, gemistocytic, small cell, and giant-shaped tumor cells showing nuclear pleomorphism are observed (H&E, ×100, ×400). (e) On histological sections, pseudopalisading necrosis is diagnostic for glioblastoma (H&E, ×100). (f) GFAP immunohistochemistry is positive in the neoplastic astroglial part whereas GFAP is negative in areas of endothelial hyperplasia (microvascular hyperplasia) (×400).

**Figure 5:**
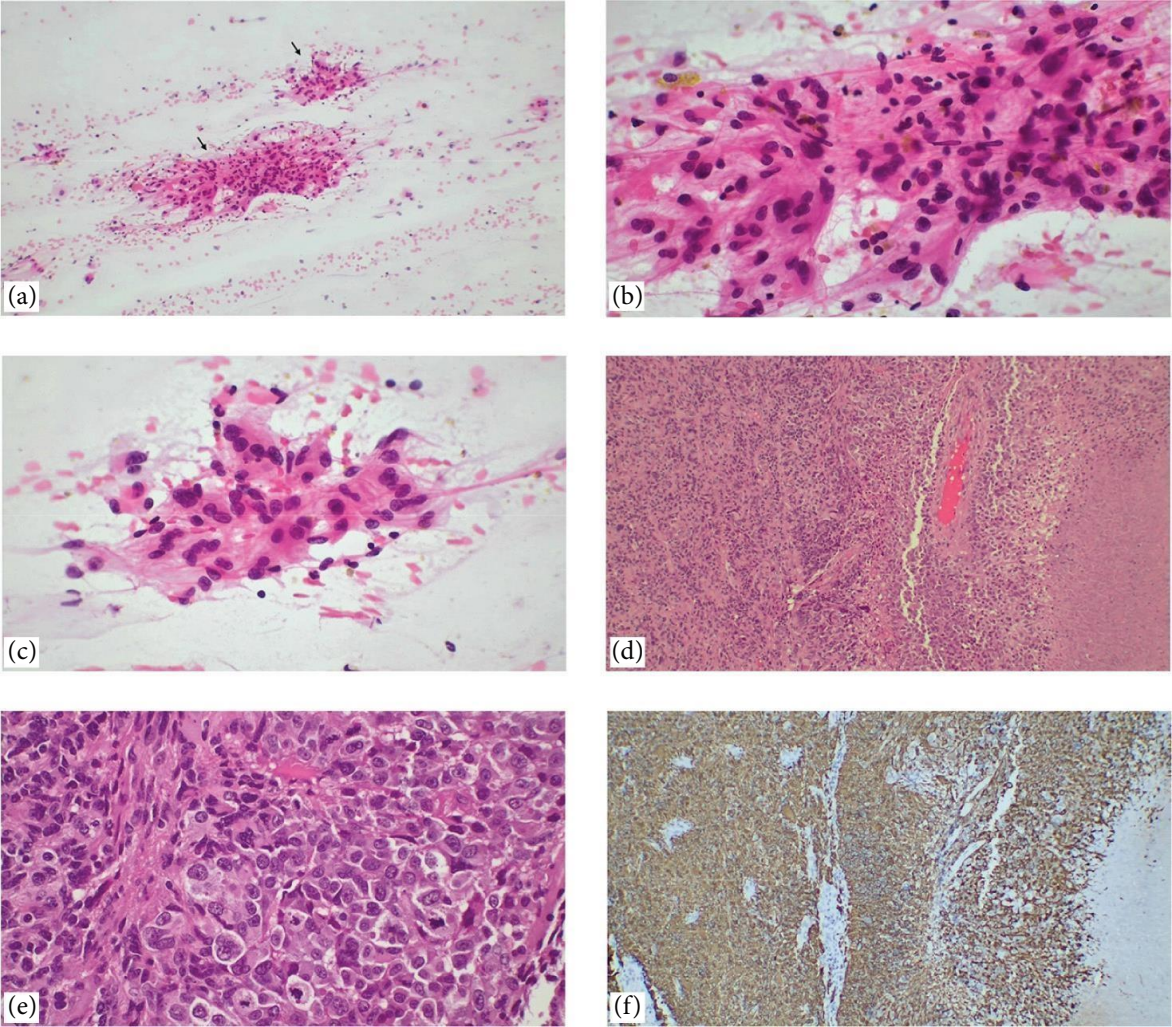
Adult-type diffuse glioma (high-grade glioma). (a–c) In touch imprint cytology, oval-elongated nuclei with marked atypia, fibrillar cytoplasmic processes, and increased cellularity are observed (H&E, ×100, ×400). (d, e) Microvascular proliferation and infarct-like necrosis are observed in paraffin sections (H&E, ×100). (f) GFAP is diffusely positive in immunohistochemical studies (×100).

**Figure 6:**
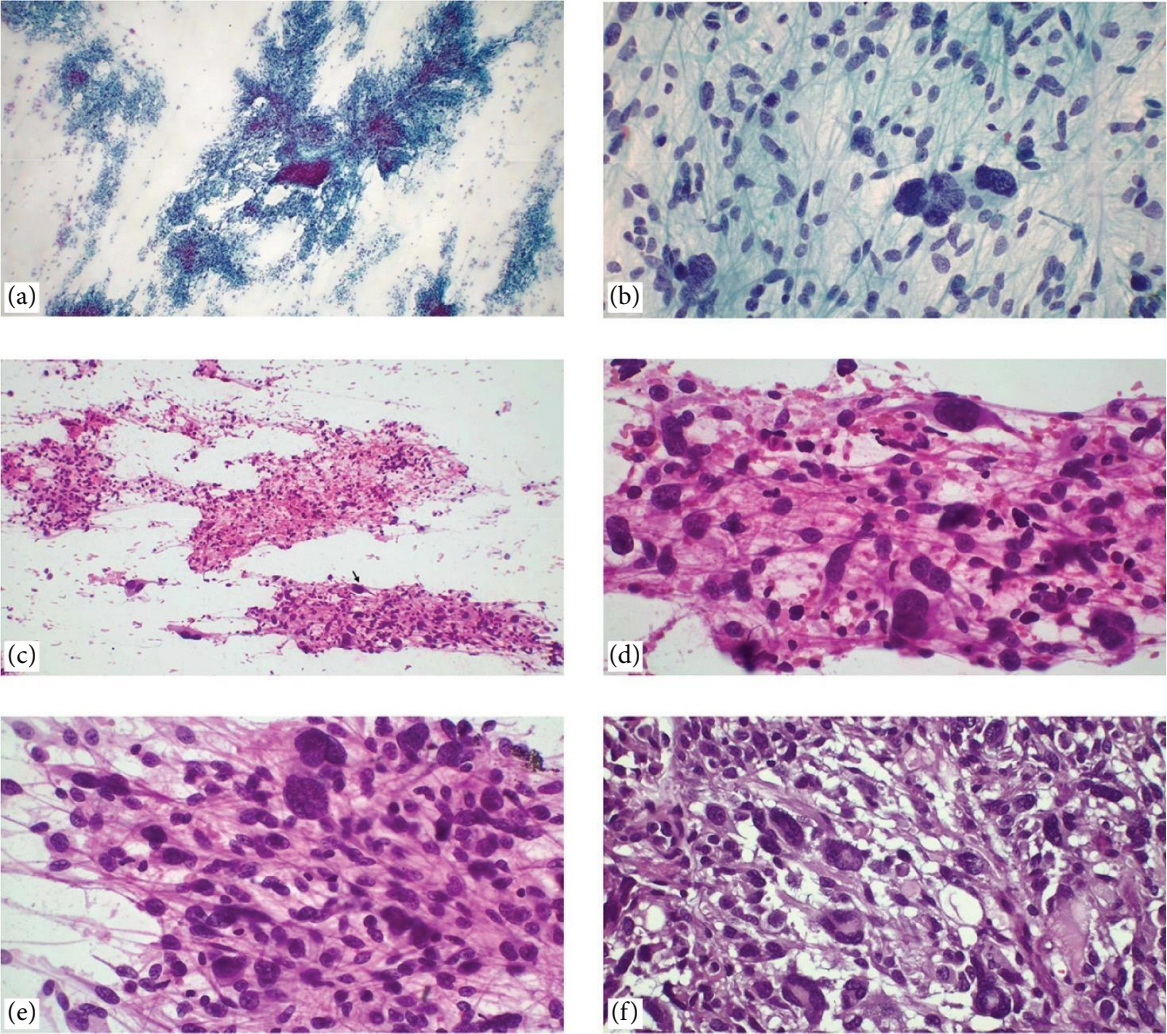
Adult-type diffuse glioma (high-grade glioma). In a patient with known malignancy, if the clinical history is reported before intraoperative consultation, high-grade glioma may be confused with suspected metastasis and the mistake may cause serious confusion. This patient had previously been treated for gastric cancer. The key points are detailed in the figures. (a, b) Papanicolaou stain, ×100, ×400 and (c–e) H&E, ×100, ×400. In touch imprint cytology, elongated, irregular hyperchromatic nuclei with prominent eosinophilic fibrillar cytoplasmic processes, and pleomorphic cells surrounded by neuropil are observed. (f) Paraffin section of the case (H&E, ×400).

**Figure 7:**
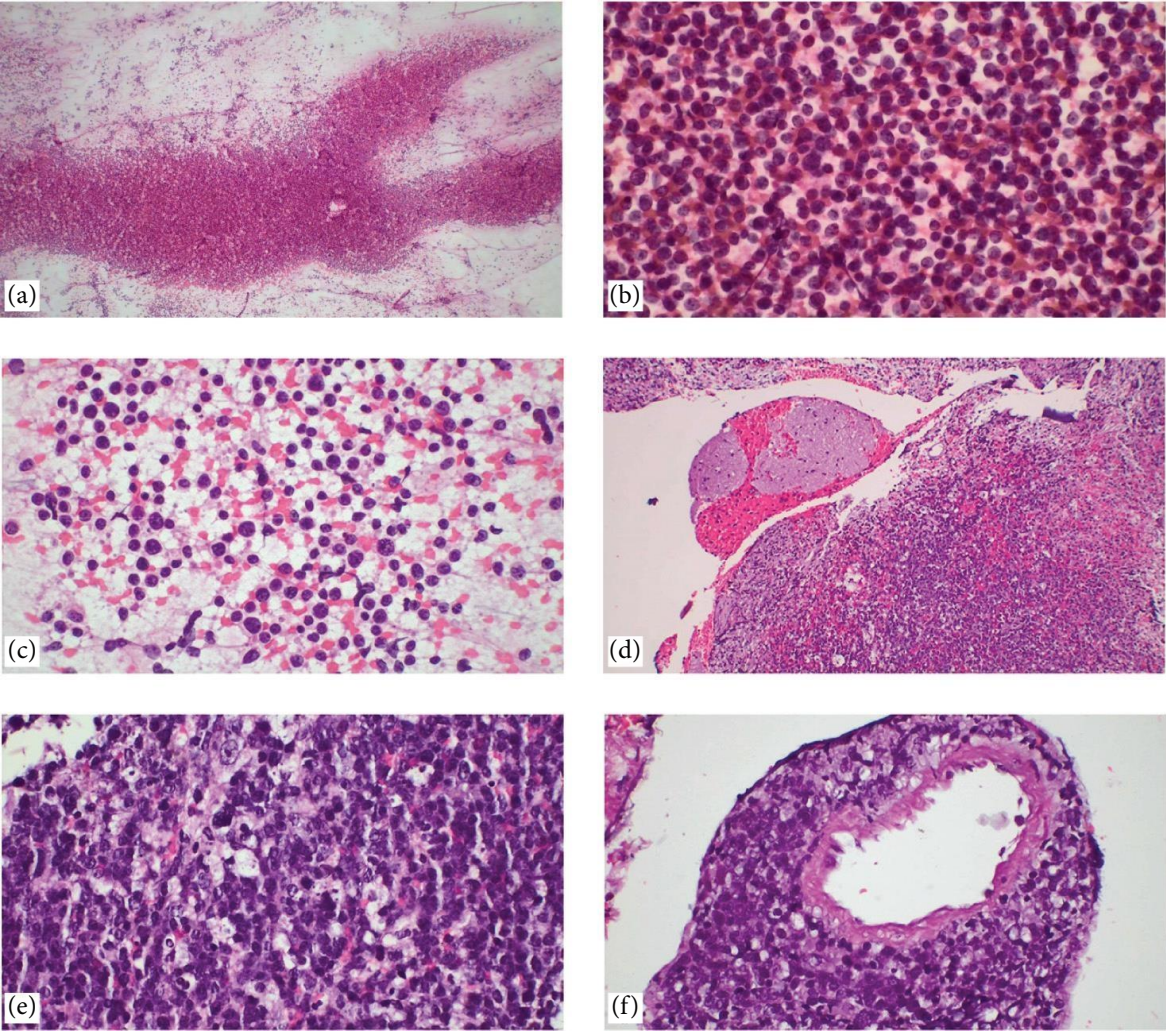
CNS lymphoma, high-grade B cell lymphoma. (a–e) In touch imprint cytology, large, lobulated tumor cell nuclei with vesicular chromatin show discohesive, scant cytoplasm, and prominent nucleoli (H&E, ×100, ×400). (d) Paraffin sections show that it is noninfiltrative to the adjacent brain parenchyma (H&E, ×100). (e) Caryorectic nuclei and cell debris are observed with typical cytological features of large B cells (H&E, ×400), (f) angiocentric pattern, including both perivascular infiltrates and angioinvasion (H&E, ×400).

**Figure 8:**
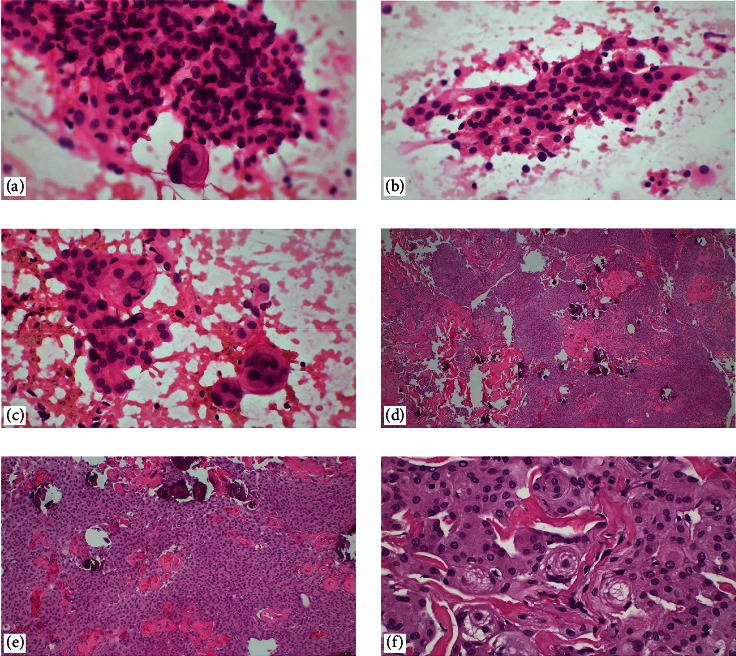
Meningioma. (a–c) Touch imprint cytology shows delicate chromatin with indistinct nucleoli, oval nuclei, and cytoplasmic pseudoinclusions and whorling (H&E, ×400). (d–f) Lobulated, nested, and whorled formations, fibrous connective tissue, and psammomatous calcifications are observed in histopathological sections (H&E, ×40, ×100, ×400).

**Figure 9:**
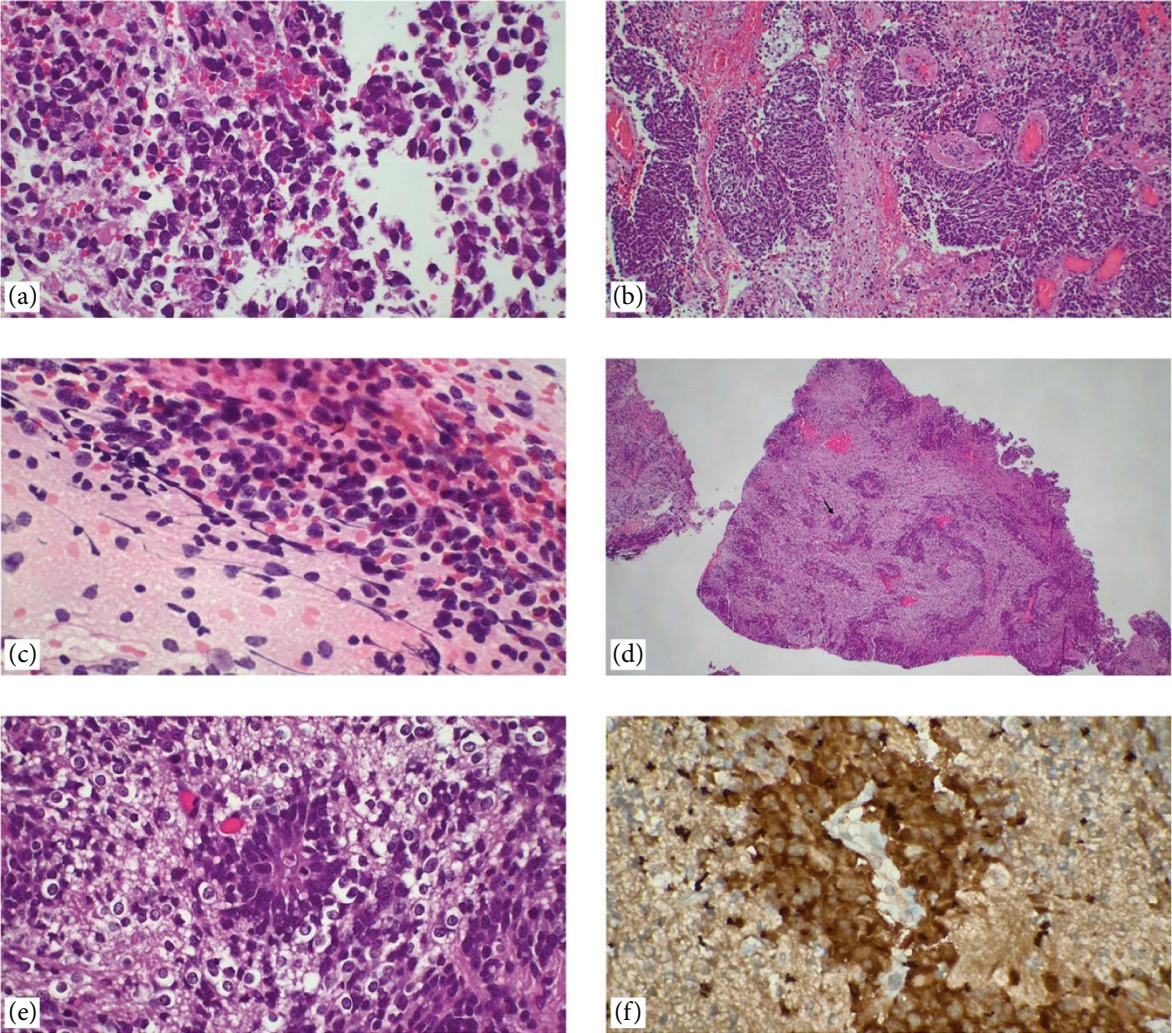
Embryonal tumors of the central nervous system. (a, b) Medulloblastoma: tumor cells are observed in groups containing hyperchromatic nuclei with minimal cytoplasm (H&E, ×100, ×400). Embryonal tumor with multilayered rosettes (ETMR). (c) In touch imprint cytology and (d) ETMRs can be used to replicate the same blast cells in multiples, biphasic histopathological pattern in low magnification; neuropil-rich areas and rosette formation are observed (H&E, ×40, ×400). (e) At higher magnification, rosettes are multilayered (H&E, ×400). (f) LIN28A has been identified as a diagnostic marker for ETMRs. ETMRs were diffuse and intensely positive in their multilayered rosettes and small blue and round cells.

**Figure 10:**
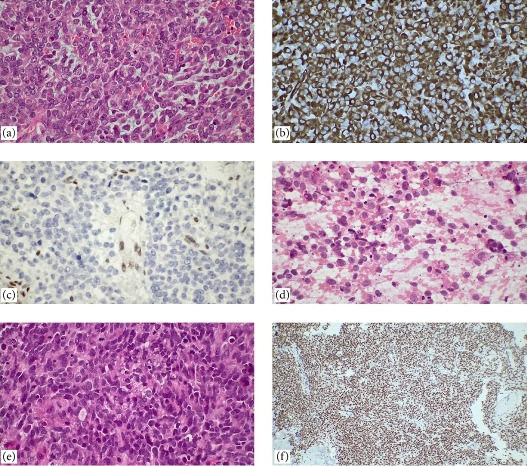
Atypical teratoid rhabdoid tumor. (a) Distinctive cell borders, large vesicular nuclei, prominent nucleoli, paranuclear inclusions with a hyaline appearance, epithelioid cells, and myxoid changes are observed (H&E, ×400). (b) Vimentin diffusely positive, (c) INI-1 loss on immunohistochemistry (×400). Metastatic BCOR internal tandem duplication of the soft tissue. (d) In touch imprint cytology, predominant round cell morphology, monotonous, undifferentiated cells with a high nuclear-to-cytoplasmic ratio, necrosis and mitotic figures, (e) histopathological sections of the case (H&E, ×400), and (f) immunohistochemically strongly nuclear positive BCOR (×100).

Rapid intraoperative consultation with cytology slides is a diagnostic tool used by some pathologists to practice neuropathology [1]. In some studies, based entirely on the pathologist's experience and the sample's representation of the lesion, a total correlation of 69%, 100% in gliomas, and 55% in metastases were established when compared with the final paraffin section diagnosis [18]. In the study of Roessler et al. [10], the diagnostic accuracy rate was 95% in cytologic smears, 97.9% in meningiomas, 96.3% in metastases, and 95.7% in glioblastomas. In our study, cytomorphological findings were consistent with the literature, resulting in a high-diagnostic accuracy rate of 94.6%. In some studies, cytohistological correlations varied between 90% and 93% [19]. However, when adequacy factors are excluded, discrepancies between the final diagnosis and the intraoperative consultation frozen section diagnosis are unavoidable. In a series of 2,156 cases, 2.7% had discrepant diagnoses. The discordant cases were found to be predominantly spindle cell lesions, oligodendrogliomas, and CNS lymphomas [7]. However, although the use of a frozen section together with cytological preparations increases the correct diagnosis rate, it is still present in those who still reach the diagnosis with a frozen section [20]. Interestingly, in another study, approximately 6% were reported as discordant and all discordant cases consisted of nonglial tumors. The majority of the errors was attributed to the inadequacy of the intraoperative consultation technique, and it was recommended to review the cases retrospectively [21]. In our study, cytomorphological findings were consistent with the literature, discrepancy was found in five cases (5.37%). In our study, the diagnosis of hematolymphoid malignancy was limited by TIC. In our study, histopathological concordance with TIC was very high in gliomas, meningiom, as and metastases, which is in accordance with the literature. The first step in the intraoperative neuropathology consultation is to determine whether there is “lesional tissue”. The first pitfalls and trapsat this stage are that normal brain tissue may be indistinguishable from tumor tissue [8, 22]. As noted above, this depends on the experience and awareness of the pathologist. Diagnostic pitfalls include choroid plexus in ventricular samples, ganglion cells in temporal cortex samples, and medulloblastoma in normal cerebellar cortex biopsies. The second pitfalls and trapsis a misinterpretation, which we call overreading, due to excessive and overlapping preparation of normal brain tissue, resulting in a hypercellular appearance. The interpretation of cellularity in TICs is a subjective feature, as the assessment depends entirely on the sampling or smear technique. The third pitfalls and trapsis the presence of nonspecific, perhaps nonneoplastic, reactive cytopathological changes that do not lead to a typical pathology, such as the presence of vascular structures containing necrosis, gliosis, or inflammatory infiltrates. In such cases, the pathologist should prefer to order a new and more representative biopsy (Figures 1(a) and 1(b)) [23]. In our study, smears with eosinophilic and granular (feltlike) backgrounds, homogeneous, without significant fibrillary, few vascular channels, and low-cellularity rate were accepted as normal neuroglial tissue. The main tumors that most frequently affect the CNS parenchyma are gliomas, glioneural tumors, and neuronal tumors [16]. Diffuse gliomas include astrocytomas, oligodendrogliomas, and glioblastomas (Figure 1(c)–1(f)), and premolecular classification diffuse astrocytomas (Grades 2–4) account for approximately 40% of all intracranial tumors, with glioblastomas being the most common [11, 24, 25]. The fourth pitfalls and traps are between radiation change involving fibrinoid necrosis of the vessel walls, telangiectatic changes, bizarre cells, and glial neoplasms, which contain macrophages and vascular channels that may be visible at the periphery of diffuse infiltrative gliomas, but generally do not show high-grade nuclear features. Fifth pitfalls and trapsare seen in the distinction between astrocytic tumors and oligodendroglial tumors. It is not recommended to make such a distinction with cytological preparations as it may require additional immunohistochemical and molecular studies. In TICs, neuropil and glial fibrillary processes are containing atypical cells with rounded bare nuclei showing hyperchromasia in the background of infiltrative astrocytomas. In oligodendrogliomas, tumor cells with round nuclei containing dense chromatin and a thin capillary vascular network are observed in a somewhat more monotonous and regular appearance. Perinuclear halos resulting from formalin fixation are not expected to be seen in TICs. In such preparations, our preference is to state that they are compatible with low (Grades 1 and 2) or high-grade (Grades 3 and 4) glial neoplasia, and the definitive diagnosis is deferred to permanent (paraffin) sections. Sixth pitfalls and traps; we report in practice that if a low-grade glial neoplasia is detected in an elderly patient, the surgical team may find a high-grade tumor in an unsampled area. It should be noted that younger cases (pediatric or young adults) may have a low-grade glial or glioneural neoplasm. Brain metastases constitute a very important burden of intracranial tumors, approximately 100,000 people are diagnosed annually, and they are reported to be 10 times more common than primary brain tumors. It has been known that approximately 18%–51% of all intracranial neoplasms are brain metastases [26]. Seventh pitfall or trap; is to distinguish between glioblastomas and metastatic carcinomasin cases with multiple mitotic figures and prominent nucleoli. In cases such as high-grade glioma, especially the small cell component of glioblastomas, cellularity may be high and glial neuropil may be less in the background, and in these cases, metastatic carcinoma may be trapped [27]. Our preference is usually for a quick review of the clinical history and radiological findings, but glial tumors are infiltrative, whereas metastatic tumors form a sharp border with the adjacent brain parenchyma (Figures 2(a)–2(f) and 3(a)–3(f)). Eighth pitfall or trap; in rim-enhancing lesions, the specimen may resembles a low-grade astrocytoma with less cellular areas sampled from the periphery of the tumor in cytological preparations, rather than the typical glioblastomas (which are generally cellular, prominent nuclear atypia, necrosis, and microvascular proliferation), and it should be reported that it does not represent a tumor (Figures 4(a)–4(f), 5(a)–5(f), and 6(a)–6(f)). The ninth pitfall or trap; is the diagnosis of neoplasms that can be observed as multiple lesions and nonneoplastic (inflammatory, vascular, infectious diseases, etc.). Because of its prognostic importance, it is important to recognize melanoma, lymphoma, metastases, and late-stage medulloblastomas as multiple lesions. The cohesive growth pattern is not usually a feature of melanoma, but the distinct border with the adjacent brain reveals its metastatic nature. Since diffuse large B-cell lymphomas are frequently observed in the CNS, lymphomas similarly show discohesive, prominent nucleoli, atypical round nuclei, lymphoglandular complex, tingible body macrophages, and (if we are lucky that day) angiocentric growth pattern (Figure 7(a)–7(f)). A few important key points in lymphomas; the absence of infiltration into the brain parenchyma, and the absence of glial processes can be counted, but in difficult cases, it may not be possible to differentiate with TIC [28]. In such cases, atypical lymphoid proliferation in lymphoid lesions, we prefer the definitive diagnosis deferred to the paraffin sections. Due to their nature, being cautious about some lesions or tumor groups is also important in diagnosis and treatment. In some lesions, the final diagnosis is predominantly deferred to permanents. Of these, atypical spindle cell process in soft tissue neoplasia, atypical spindle cell proliferation in fibrous meningiomas are given during intraoperative consultation, frozen sections do not always contribute to the diagnosis due to superimposed artifacts, cautery, physical crush, thick sections, and drying [28]. Tenth pitfall or trap; they are spindle cell lesions that are included in the diagnosis and differential diagnosis of nonglial tumors such as meningioma, schwannoma, and neurofibroma. A few key points in such lesions; if we consider a high-grade (atypical/anaplastic) meningioma or if we suspect a soft tissue tumor such as a solitary fibrous tumor, we deferred the grade or final diagnosis to paraffin sections. Intranuclear cytoplasmic pseudo inclusions are characteristic in meningiomas but can also be observed in melanomas. Detection of meningothelial whorls and calcifications in a classic meningioma is straightforward (Figure 8(a)–8(f)). The eleventh pitfall or trap; is the distinction between embryonal tumors, germ cell neoplasms, and hematolymphoid malignancies. In particular, medulloblastoma (Figure 9(a)–9(f)) and atypical teratoid/rhabdoid tumor, soft tissue sarcomas can be confused with small cell carcinomas, neuroblastoma, and lymphomas, while embryonal tumors with multilayered rosettes can be confused with lymphomas and anaplastic ependymoma (Figure 10(a)–10(f)) [29, 30]. As in all areas of pathology, there are potential pitfalls in the intraoperative neuropathology. These pitfalls are numerous and new classifications, new molecular discoveries or definitions have not put an end to these pitfalls. Even molecular studies may create new potential pitfalls. The confusion of a low-grade glial tumor with reactive gliosis in the brain parenchyma adjacent to the tumor and the potential entrapment of anuclear squamous nodules are pitfalls. Another pitfall is that granulomas occasionally encountered in gonadal germinomas are diagnosed as a granulomatous inflammatory process, especially in small biopsies. In this study, we have generally mentioned the cases and pitfalls we have encountered in our own practice. However, among the potential pitfalls, the frequency of mitosis and WHO histological grading may pose a problem in the grading of meningiomas and in the recognition of infiltrating gliomas [17, 22, 28, 31]. One of the problems we encounter in our practice is the grading of gliomas. The main reasons for this are inadequate sampling and lack of cytomorphological features. In general, the grade of cytological sampling may be lower than the grade of routine paraffin sections. For example, the distinction between a low-grade, well-differentiated astrocytoma, and anaplastic astrocytoma may not always be possible.

In planning for CNS tumors, both pathologists and surgical oncology clinicians should be aware of the limitations of intraoperative consultation due to technique and pathologist experience. A limitation of our study is that it is single-center and the possibility of regional differences may not represent the true distribution of patients. However, our sample size was large sufficient to make the results of our study meaningful. Another limitation of our study is that the specimens were not evaluated contemporaneously by an inexperienced pathologist. One of the problems encountered in the intraoperative diagnosis of such CNS specimens was that inexperienced assistants (due to training) were not able to apply the TIC technique adequately, resulting in failure to achieve the qualification criterion of high cellularity. Another limitation is the presence of too many bakcground artifacts such as poor cellularity, necrosis and hemorrhage, drying artifacts, and excessive fibrous tissue samples. Another limitation of the procedure is that the tumor may be completely consumed during these procedures and the pathologist may have to spend more time than necessary.

The classification of CNS tumors, especially gliomas, has changed significantly with the advancement of molecular techniques [32]. In the 2016 WHO update, the classification of adult-type diffuse gliomas was based on IDH mutation, and astrocytomas and oligodendrogliomas were subclassified by determining the TP53, ATRX, 1p/19q codeletion status, while diffuse midline gliomas were defined with the H3K27M mutation [33]. In the 2021 WHO update, new additional molecular studies were developed for adult-type gliomas. These include H3.3 Histone A (H3.3A) mutation, telomerase reverse transcriptase (TERT) promoter mutation, epidermal growth factor receptor (EGFR) gene amplification and chromosome seven gain combined with loss of chromosome 10, and homozygous deletions of both cyclin dependent kinase inhibitor 2A (CDKN2A), and cyclin dependent kinase inhibitor 2B (CDKN2B). A multi-gene panel (Glio-DNA panel) was developed for glioma oncotypes using the recent next-generation sequencing (NGS) molecular technique. In this study, important results were obtained regarding the correct classification of gliomas, detection of genomic anomalies, and identification and detection of mutations in druggable genes for target treatments in glioma patients [34].

## 5. Conclusions

TIC is a rapid, safe, and inexpensive diagnostic tool used during intraoperative consultation in the neuropathology. There are some limitations and important pitfalls in the use of this technique. In our study, the use of imprint cytology alone showed high sensitivity and specific diagnostic accuracy. Our results also provided examples of important touchstones for possible pitfalls to be encountered during intraoperative consultation and contributed to the experience and training in neuropathology.

## Figures and Tables

**Table 1:** Patient demographics and histopathologic characteristics.

General category interpretation	Number of cases (%)	Sex (M/F)	Median age (years)	TIC + SP + biopsy/only biopsy/cytology (%)	Correct diagnosis; misdiagnoses/accuracy = *n*
A-neoplastic-primary tumors					
A.1-Glial/glioneural tumors or lesions	—	32/16	48.8 (±20.8)	37 (77.1)/11 (22.9)	—
Glioblastoma, IDH-wild type, Grade 4	24 (16.7)	—	—	—	1/15
Astrocytoma, IDH-mutant, Grade 4	1 (0.7)	—	—	—	—
Astrocytoma, IDH-mutant, Grade 2	3 (2.1)	—	—	—	0/3
Astrocytoma, Grade 2	7 (4.9)	—	—	—	0/7
Pilocytic astrocytoma, Grade 1	4 (2.8)	—	—	—	0/4
Oligodendroglioma, IDH-mutant, Grade 3	2 (1.4)	—	—	—	0/2
Pediatric-type diffuse high-grade gliomas	1 (0.7)	—	—	—	—
Spinal ependymoma	1 (0.7)	—	—	—	0/1
Supratentorial ependymoma, Grade 3	1 (0.7	—	—	—	0/1
Gliosis	2 (1.4)	—	—	—	0/1
Radiation-induced change	1 (0.7)	**—**	—	—	0/1
Cortical tubers	1 (0.7)	**—**	—	—	0/1
** **A.2-Nonglial tumors or lesions	—	20/28	49.9 (±19.8)	11 (22.9)/37 (77.1)	—
Meningioma, meningothelial, Grade 1	10 (6.9)	**—**	—	—	0/4
Meningioma, fibrous type, Grade 1	5 (3.5)	**—**	—	—	0/5
Meningioma, psammomatous, Grade 1	1 (0.7)	**—**	—	—	0/1
Meningioma, angiomatous, Grade 1	2 (1.4)	**—**	—	—	0/1
Atypical meningioma, Grade 2	4 (2.8)	**—**	—	—	0/4
Schwannoma, Grade 1	4 (2.8)	**—**	—	—	0/2
Neurofibroma, Grade 1	1 (0.7)	**—**	—	—	0/1
Hybrid nerve sheath tumor, Grade 1	1 (0.7)	**—**	—	—	0/1
Hemangioblastoma, Grade 1	2 (1.4)	**—**	—	—	1/0
Pituitary adenoma	10 (6.9)	**—**	—	—	0/1
Pituitary apoplexy	2 (1.4)	**—**	—	—	0/1
Hydatid cyst	1 (0.7)	**—**	—	—	0/1
Hemangioma	2 (1.4)	**—**	—	—	0/1
Medulloblastoma, non-WNT/non-SHH, Grade 4	1 (0.7)	**—**	—	—	—
Embryonal tumor with multilayered rosettes, Grade 4	1 (0.7)	**—**	—	—	0/1
Atypical teratoid/rhabdoid tumor, Grade 4	1 (0.7)	**—**	—	—	—
B-Neoplastic-metastatic tumors	—	32/16	55.4 (±13.6)	31 (64.6)/15 (31.3)/2 (4.2)	—
Metastatic lung carcinomas	19 (13.2)	—	—	—	0/14
Metastatic breast carcinoma	8 (5.6)	—	—	—	0/3
Metastatic colorectal carcinomas	3 (2.1)	—	—	—	0/2
Metastatic (other) gastrointestinal carcinomas	3 (2.1)	—	—	—	0/3
Metastatic clear cell renal cell carcinomas	1 (0.7)	—	—	—	0/1
Metastatic thyroid carcinomas	2 (1.4)	**—**	—	—	0/1
Metastatic ovarian carcinomas	2 (1.4)	—	—	—	0/1
Metastatic carcinoma of unknown primary	1 (0.7)	**—**	—	—	—
Metastatic melanoma	2 (1.4)	—	—	—	—
Hematolymphoid neoplasm	6 (4.1)	—	—	—	3/2
Metastatic sarcomas	1 (0.7)	—	—	—	0/1
Total cases	144 (100)	—	—	—	5/88

F; female, M; male, TIC; touch imprint cytology, SP; squash preparations.

**Table 2:** Sensitivity and specificity of touch imprint cytology and final histopathological diagnosis for brain neoplasms.

	Positive/total (%)		
	Glial tumors	Nonglial tumors	Metastatic (secondary) tumors
Sensitivity	32/33 (97)	20/21 (95.2)	28/31 (90.3)
Specificity	4/4 (100)	4/4 (100)	3/31 (9.7)
Positive predictive value	32/32 (100)	20/20 (100)	28/28 (100)
Negative predictive value	4/5 (80)	4/5 (80)	3/3 (100)

Sensitivity = true positive/true positives + false negatives; specificity = true negatives/true negatives + false positives; positive predictive value = true positives/true positives + false positives; negative predictive value = true negatives/true negatives + false negatives.

**Table 3:** Results of a diagnostic TIC presented.

Result of diagnostic TIC	Results of routine paraffin sections	
	Tumors positive	Tumors negative
TIC positive	80	0
TIC negative	5	8

**Table 4:** Correlation of touch imprint cytology and final histopathological diagnosis.

Cytologic diagnosis	Histopathologic diagnosis	Total number of cases
High-grade glioma	Glioblastoma, Grade 4	15
Nondiagnostic samples	Glioblastoma, Grade 4	1
Low-grade glioma	Astrocytoma, Grade 2	10
Low-grade glioma	Pilocytic astrocytoma, Grade 1	4
High-grade glioma	Oligodendroglioma, Grade 3	2
Low-grade ependymoma	Ependymoma, Grade 1	1
High-grade ependymoma	Ependymoma, Grade 3	1
Nonneoplastic and hamartomatous lesions	Gliosis, radiation-induced change, cortical tubers	3
Meningioma	Meningioma, Grade 1	11
Meningioma	Meningioma, atypical Grade 2	4
Spindle cell lesions	Schwannoma, neurofibroma, hybrid nerve sheath tumor, Grade 1	4
Nondiagnostic	Hemangioblastoma, Grade 1	1
Adenoma	Pituitary adenoma/apoplexy	2
Benign; vascular neoplasia	Hemangioma	1
Nonneoplastic, granulomatous inflammatory processes	Hydatid cyst	1
Small blue cell tumor	Embryonal tumor with multilayered rosettes, Grade 4	1
Metastatic, epithelial	Carcinoma	25
Atypical lymphoid proliferation	Hematolymphoid neoplasm	3
Malignant lymphoma	Diffuse large B-cell lymphoma (DLBCL)	2
Malignant, spindle cell lesion	BCOR internal tandem duplication of the soft tissue	1
Total	—	93

## Data Availability

The data supporting this study's findings are available from the corresponding author upon reasonable request.
